# A “messy ball of wool”: a qualitative study of the dimensions of the lived experience of obesity

**DOI:** 10.1186/s40359-020-00416-2

**Published:** 2020-06-25

**Authors:** Kathryn Ogden, Jenny Barr, Georgia Rossetto, John Mercer

**Affiliations:** 1 Tasmanian College of Medicine, University of Tasmania, Locked bag 1377, Launceston, Tasmania 7250 Australia; 2 Launceston Dietetics, 5 Innes Street, Launceston, Tasmania 7250 Australia; 3 Department of Allied Health, Launceston General Hospital, Tasmanian Health Service, Launceston, Australia

**Keywords:** Embodiment/bodily experiences, Health care, Lifeworld led care, Lived experience, Obesity, Psychology

## Abstract

**Background:**

Obesity is a multi-dimensional condition with causal factors beyond the physiological into the behavioural, dietetic and psychological. Understanding the lived experience of those who are overweight and obese and self-perceived barriers to access and engagement in intervention are imperative to formulating a systemic response to the complex problem of obesity. This study aims to identify the social, psychological and systemic factors impeding engagement with weight-loss behaviour and interventions, and to formulate a framework for responding to these.

**Methods:**

We conducted an exploratory qualitative study using focus groups and interviews with people who have lived experienced of being overweight or obese. Data were analysed using an inductive thematic approach. Following the thematic analysis, further interpretation of the data was achieved by applying the epistemological foundations of the Lifeworld Led Care paradigm, recognising its philosophy of the person and of care based on the individual’s experiences. Eight men and 17 women participated.

**Results:**

Three overarching themes were identified: Complexity and Battle, Impediments, and Positive Re-orientation. The subthemes of these were found to represent the dimensions of the Lifeworld: Identify, Inter-subjectivity, Mood and Embodiment.

Further interpretation of the themed data identified six polarised dichotomies representing the opposing lived dimensions of the obesity experience: Failure Double-Bind; Think-Feel Conflict; Negative-Positive Orientation; Impeding-Facilitating Health Professional; Knowledge as Deficit-Insight; and Internal-External Orientation.

**Conclusion:**

Obesity manifests as constraints and challenges across six polarised dichotomies, active in the lived experience of obesity. This study provides a unique way of conceptualising and understanding the complex and interacting meanings of the lived experience of obesity through the construction of polarised dichotomies. The polarities signify the oscillating experiences that people with obesity encounter, which may be either helpful or destructive in both their lifeworld experience and their capacity to address obesity towards improved social, psychological and physical outcomes. Understanding the dichotomies allows a reconceptualisation of obesity from a quantification of the individual to a more respectful, humane, compassionate and utilitarian conceptualisation of the experiencing person and the phenomenon itself. Further, these lived polarised dichotomies of obesity present the opportunity for health professionals to reconceptualise obesity in care and interventions.

## Background

As a chronic condition with a high burden of illness [1, 2], obesity has been subject to extensive quantitative analysis, and outcomes from obesity interventions are usually measured in quantifiable terms such as Body Mass Index. However, obesity exhibits multidimensional causal factors extending beyond diet and physiology to behaviour and psychology [3–7]. The majority of individuals fail to achieve sustained weight loss solely from calorie-control interventions [8] and people are likely to benefit from different forms of weight loss intervention [9].

More than a decade ago, Thomas et al. [5] identified diverse experiences of obesity, but with common themes, highlighting the need to tailor interventions to individual needs, and ensure damaging stereotypes are avoided. This study highlighted the need to rethink how to approach obesity without perpetuating damaging stereotypes at a social level, in addition to the specific needs of individuals. However, the medicalisation of obesity [10] has led to efforts to address obesity focussing on a medical model of management, with guidelines focussing on lifestyle modification, behaviour therapy, pharmacological and surgical intervention [11–13], and the ultimate outcome measured quantitatively. Recent publications have sought to bring back the focus of lived experience as relevant to how obesity is managed by the health care sector. Ueland et al. [14] identify objectification as impeding progress, and a need for health care workers to assist persons living with obesity to reduce objectification and alienation through interventions that have an individual holistic approach. Haga et al. [15, 16] identify complex existential experiences which provide deeper insights into the lived experiences of people with obesity, that can inform a more comprehensive approach to obesity health care.

Understanding patient-centred experience and outcomes in obesity care may require an expanded focus on inter-related elements of the obesity experience. Doing so will allow representation of the full complexity of obesity and more adequately frame evidence pertaining to obesity as a “lived” condition which aligns with patient-centred care [17]. Although a number of models have been developed in attempts to profile different aetiologies of obesity on the basis of character and behaviour [18, 19], the application and evaluation of such models during assessment and treatment of obesity is underdeveloped. The use of such approaches towards developing pragmatic, evidence derived guidance to health professionals’ interactions with people experiencing obesity is lacking.

Research that is not focussed on what concerns the person with a lived experience of obesity potentially reduces obesity programs and health interventions to kilogram focussed outcomes, diminishing the understanding of the health and wellbeing of the person with obesity [17] and contributing to further stigmatisation. Tomiyana et al. [20] comment on the prevalence of weight stigma in healthcare settings, across both healthcare provider attitudes as well as provision of care, and call for a change to health provider training for a different understanding of the effects of bias on patients.

There is evidence to suggest that people who experience obesity benefit from psychological interventions particularly when implemented in conjunction with exercise and/or dietary control [21–23]. For example, in bariatric surgery populations, post-surgical lifestyle intervention can improve weight loss outcomes [24], however the psychological variable of control has been found to be a key factor for poor post-surgical adjustment and outcomes [25]. Understanding peoples’ lived experience of obesity [5, 6, 26] and their self-perceived barriers to access and engagement in intervention are imperative to formulating a systemic response to the complex problem of obesity that involves health provider training as well as care delivery.

This study is aimed to be an in-depth exploration of the lived experience of obesity in line with the lifeworld approach described by Todres, Galvin and Holloway [27], with the overarching aim to inform the further development of tailored interventions, guidelines for health professional training and to address restraints to treatment engagement. To achieve this aim, we explored ways in which effective lifestyle change and weight loss may be influenced by: the physical and psychological impact of the condition; the potential motivating and facilitating factors; impediments to successful intervention; intrapersonal factors; external influences; and patients’ expectations of an ideal weight loss program.

## Methods

### Theoretical framework

The orientation for this study was informed by the Lifeworld-Led Care [27–31] paradigm for qualitative health research. Lifeworld-Led Care (LLC) is relevant to research into lived experience across complex and chronic health domains, bringing together fundamental aspects of phenomenology, existentialism, ontology and hermeneutics, which reconceptualises the ‘lived’ place of the person in complex health care systems [32].

Dahlberg, Todres & Galvin summarise the core components of the LLC paradigm as “a philosophy of the person, a view of well-being and not just illness, and a philosophy of care that is consistent with this” ([28, p. 265]). They emphasise that their philosophy of the person is existential, and that this existential understanding of the human being is the foundation for a phenomenological Lifeworld understanding of well-being and illness. The existential dimensions of the Lifeworld - temporality, spatiality, inter-subjectivity, embodiment, mood and identity – are at the centre of LLC [30, 33, 34].

### Overarching methodology

We conducted an exploratory qualitative study drawing on both focus groups and individual interviews of people who have experienced overweight or obesity. The investigation team was a multi-disciplinary group consisting of a general practitioner/academic researcher, patient-centred care academic researcher, dietitian and a chronic condition psychologist.

### Recruitment and sample

We used purposive sampling to assemble a sample of participants able to contribute to the aims of the research. Our initial recruitment strategy involved an invitation to general practices inviting referrals, however this failed to be a successful mechanism. Consequently, it was decided to recruit directly from two lifestyle intervention programs for people concerned about excess weight as well as a patient partnership education program which involved people with chronic illness relating to being obese. We identified a 12-week program which primarily focused on physical activity, but also provided sessions for dietary advice and support, in addition to a 6-week program focussing on a psycho-dietetic approach to managing eating behaviour. Participants in these programs self-identified as people with obesity, they were interviewed prior to or in the very early stages of these programs. Further recruitment came from people in the community with chronic illness who were engaged in an undergraduate medical education program, the Patient Partner Program [35]. Two participants were recruited opportunistically by a colleague outside of these programs.

The key criteria for inclusion was that participants perceived a need for them to lose weight to improve their health and well-being. The rationale for this related to the clinical perspective of researchers and a desire for the research to inform a clinical response to obesity. We did not limit participation based on anthropometric measures; however, our recruitment strategies were aimed at avoiding recruitment of individuals whose weight loss desire was unrelated to health and well-being issues. This was possible as a result of the knowledge we had of the programs from which participants were recruited, which all had a focus on people with obesity that related to health and well-being concerns. We did not screen for eating disorders. Participants were required to be able to speak proficient English and be over the age of 18.

### Interview methods

Focus groups and interviews were facilitated by two of three authors (KO, JB, GR) on all but one occasion, with an experienced qualitative researcher (KO or JB) present for each. One focus group was conducted by KO alone. Interviews were semi-structured with the natural flow of conversation allowed, but using the prompts contained in the interview schedule to ensure all research questions were addressed (Additional File, Item 1). During the focus groups, discussion between participants was encouraged, to develop a rich discourse and deep thinking by participants, prompted by researchers using the interview schedule. Facilitators ensured that all participants were able to express their experiences and thoughts.

### Data analysis

Audio files of focus groups and interviews were transcribed verbatim and analysed using an inductive thematic approach. The aim was to provide a rich thematic description of the dataset to gain an understanding of the broad range of experiences, meanings and reality of our participants [36]. This approach allowed us to: condense raw textual data into a brief, summary format; establish clear links between research objectives and the summary findings derived from the raw data; and develop a framework of the underlying structure of experiences or processes that are evident in the raw data [37]. NVivo 10 software [38] was used to support data analysis and management. Raw data were analysed by three authors (KO, JB, GR) who identified themes and subthemes independently. Final themes and their relationship to each other were agreed on by all authors collaboratively. While the analysis was data-driven, our personal and clinical experiences and knowledge of patient-centred care are acknowledged as is pertinent with qualitative research [36].

To determine the point of data saturation, one author (KO) thematically analysed the data after the first 13 participants (2 focus groups and 3 interviews) and was present for all subsequent focus groups and interviews so was able to continually monitor whether new themes were emerging. Theoretical sampling and cohesiveness of the sample led to a rich and complete dataset, albeit requiring consideration of external validity [39]. Information power was aided by the relatively narrow aims of the study, sample specificity, use of theory, and rich quality of dialogue gained from the interview and focus group methodology [40]. Data saturation was further confirmed at the analysis stage by two additional researchers (JB, GR).

Following the inductive thematic analysis, an interpretive process was undertaken to theorize the significance of the themes into broader meanings and implications [36]. The LLC framework was applied and a cluster of six distilled, polarised dimensions emerged. This interpretation of meanings and understandings was conducted by one author (JM), with confirmation by the other authors. Gender differences were not investigated as this was not a stated objective of the research.

## Results and interpretation

A total of 26 participants were involved in the study, eight men aged between 26 and 77 years (median 54.5 years) and 18 women aged between 33 and 69 (median 56) (Table 1). Two focus groups were conducted with female participants only, four in one group (FG1, ages 33–69) and six in the other (FG2, ages 45–69). One focus group was conducted including all eight men (FG3, ages 26–77). The remainder of participants were interviewed. The participant group was a relatively underprivileged group, with 15 participants in possession of a government health care card (a proxy measure for financial disadvantage in Australia) or receiving a pension; seven were in part- or full-time work and all were white Anglo-Saxon.

**Table 1:** Participant demographics

Participation type	N sex (age)
Focus group 1 (FG1)	Four females (33, 42, 55, 69a)
Focus group 2 (FG2)	Six females (45, 55, 60, 61, 63, 69b)
Focus group 3 (FG3)	Eight males (26, 39, 42, 46, 63, 69, 71, 77)
Interview	Seven females (32, 39, 48, 56, 61, 66a, 66b) One male (63)

### Thematic analysis

**Fig. 1:**
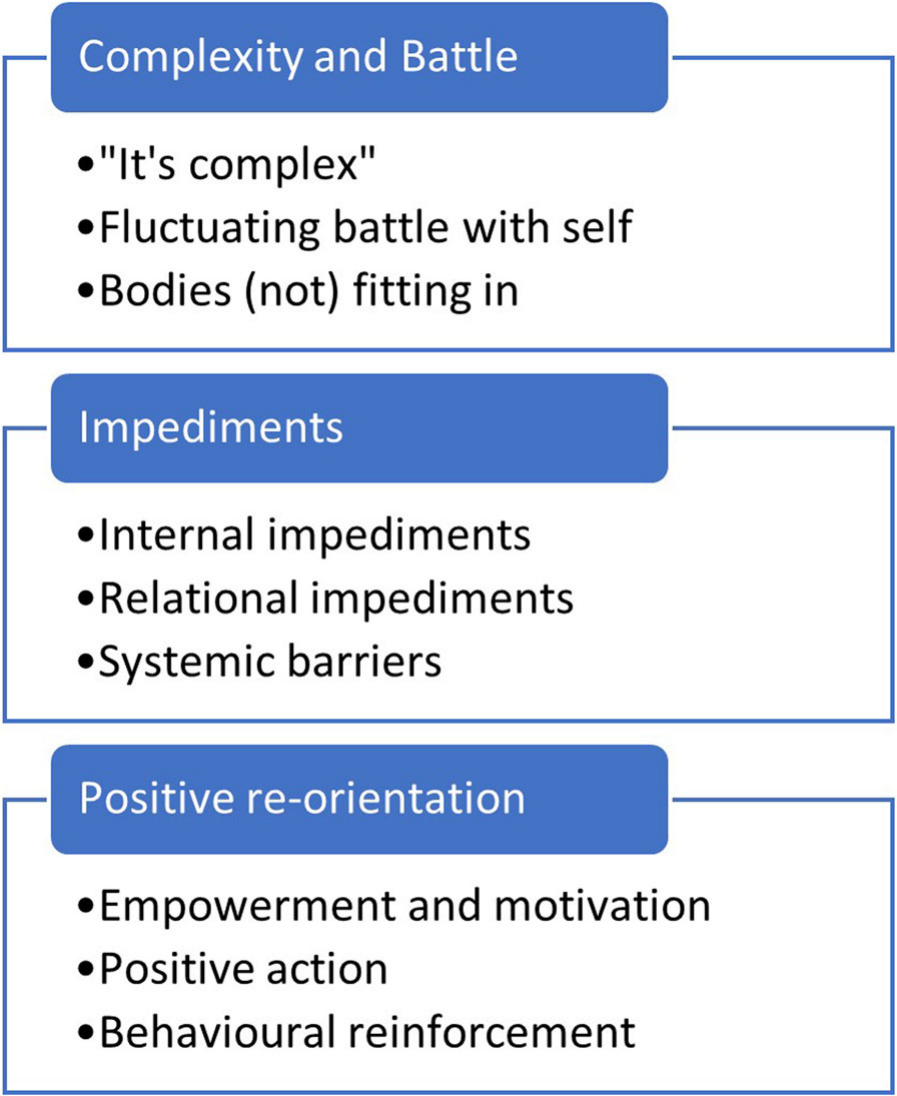
Overarching themes and subthemes derived from thematic analysis

A framework of participants’ experiences was determined as three overarching descriptive themes: Complexity and Battle, Impediments, and Positive Re-orientation, each with three subthemes (Fig. 1). Each theme is explored in Table 2, with supporting extracts from transcripts.

**Table 2:** Description of themes and subthemes with supporting quotes

Theme 1: Complexity and battle *“It’s a messy ball of wool, really.” (FG2, Female 45)* The experience of overweight and obesity was painted as a complex picture with many interacting parts, often interacting in unpredictable ways, with difficulty in finding a rhythm that allowed desired action to be taken. Challenges were both emotional and physical, with the deviation from a societal norm adding to life’s challenges.		
It’s Complex	A complex interplay of factors was at play which both precipitated and perpetuated difficulties in addressing obesity. It felt like a “vicious cycle” or a “catch 22.” Frustration occurred as participants could identify the challenges and what needed to be done but could not explain their own behaviour, which sabotaged their efforts. This confusion prompted acknowledgement that there was no silver bullet, it wasn’t a linear process, and there was danger in considering interventions such as bariatric surgery in this light. Understanding this complexity translated to knowing that a unidimensional approach could not be successful.	*If I knew probably what the barriers were, I wouldn’t be struggling so much... I should be old enough and wise enough to say “that can stay in there till tomorrow night” but no I’ve got to demolish it . . . I think I know I can’t do that, but I should, but I can’t, but I should, it’s very conflicting. (Female 48)* *The worse you eat the worse you feel. (Female 32)* *We order the food in and we think, yes this will fix me because we have someone dealing with my food but we forget that no one is dealing with the exercise, no one is dealing with our mental health, no one is dealing with the cleaning and no one is dealing with the 100 billion other jobs we need to do. (FG1, Female 42)* *. . . looking back on it now I think I was looking at it [bariatric surgery] as a way to solve all of my problems . . .pinning all my hopes on the fact that this was going to fix everything . . . I don’t think it does that at all. (Female 32)*
Fluctuating battle with self	It was difficult to find balance in order to focus on spending time on weight loss management. Personal capacity and psychological challenges presented as hurdles, creating a sense of struggle, impeding change and limiting hope. A sense of angst occurred when personal ‘wins’ or visible results were absent leading to a vicious cycle and greater hurdles to overcome. Physically, being overweight caused everything to be more difficult - leading to a feeling of battle with one’s own body. Motivations fluctuated, with conflict between wanting to lose weight but lacking motivation. Despite these challenges there was also a desire for this battle to be overcome regardless of how tough it was. Inevitably there were ebbs and flows, fluctuations in the battle, wins and losses, positive and negative experiences. The battle was all-encompassing.	*I thought maybe it's something in my mindset . . . like a barrier . . . the light at the end of the tunnel is pretty dull. . . . [It’s a] physical strain carrying around this much weight. (Female 56)* *I want to yeah to get the battle fought and won, to a place where I can be content with my weight . . .finding that balance meeting everyone else’s needs and time for me really hard. . . . to see the results you need to work hard you know um so if you don’t see the results straight away you fall off the wagon. (Female 32)* *. . . it takes determination day in and day out, why doesn’t it work. . . . there is a difference between wanting to lose weight and being motivated to lose weight … I am not always motivated to lose weight, sometimes I don’t give a stuff. (Female 66a)* *I struggle with motivation. I can get really really motivated and then the littlest thing will get me off that track and I will make all the excuses under the sun to to get off track and then getting back on track is really hard. (Female 59)* *Words depicting a herculean effort are used: “fight,” “win,” “determination,” “tough,” “hard work,” “overwhelming.”* *“It’s a yo-yo battle,” “rollercoaster,” “spiralling,”, “plateau,” “tunnel-like.”*
Bodies (not) fitting in	A further manifestation of the battle is the experience of being restricted, trapped, a body being squashed into a space. Normal activities such as buying clothes, cutting toenails, intimate relations, are impacted by size. Even the feeling of clothes can be a reminder of the attached stigma; accommodating regular changes in size with weight fluctuation also a consideration Fear about ‘fitting into daily life’ resulted in limitations on participation in activities, with obesity or size accentuating the isolation experience and further impacting on interpersonal relationships. The design of the physical environment contributed to negative experiences, compounding the impact of obesity itself.	*I’m trapped in the house I can’t get out I’m just trapped there… (Female 59)* *I just want to get home and take my clothes off because they are too tight. (FG1, Female 69a)* *…sorry we don’t have anything in your size dear, you know, before you have even tried anything on or looked at anything. (Female 56)* *Well general discomfort carrying this, when I bend over to paint toenails or even to do up my shoes I start to feel quite strange. . . . it impacts on my sex life because there is this great big gut hanging there. (FG2, Female 69b)* *I’m scared of my weight if I go out somewhere, you know they have got plastic tables and chairs and I’m scared to sit on a plastic chair because on one occasion, I actually had a chair collapse underneath me… there are times when say my friends wanted to go to the movies I didn’t want to go because I physically couldn’t fit in the seat. (FG3, Male 39)* *One of the things I have had to work with is the fact that my trousers, I had to try and find a trouser that will accommodate the fact that I am losing weight. . . we worked out that there is a particular design that has tabs on the side. (Female 42)*
Theme 2: Impediments *“I have tried so jolly hard to lose it and met with so many brick walls.” (Female 56)* *“. . . it’s just a very, very hard road.” (Female 47)* Impediments were pervasive for most participants, as indicated by the three sub-themes: internal, relational, or structural. Impediments led to a negative orientation, making taking action to improve health outcomes challenging.		
Internal impediments	Internal impediments relate to the challenges which participants believed they should have control over them but didn’t. Understanding why a level of control could be exercised in other aspects of life, but not this, was perplexing. A high level of psychological involvement was identified in weight related health behaviours. This was articulated in different ways with descriptions of the psychological aspect as emotions, mindset, in the head, attitudes and a “psychological thing.” This psychological element created barriers to change. There was a desire to behave in one way, however actual behaviour could be the opposite. Understanding that behaviours were contributing to weight but feeling powerless to stop them, or not knowing how to change, was frustrating. Furthermore, this led to negative self-perceptions which also impeded change. This feeling of lack of psychological control was compounded for those who found emotional solace in eating. Habituation was more common-place than compulsivity, however there was movement in and out of ‘habits’. Coming off diets was an example of a retreat back to previous habits, as were shopping behaviours. Habits provided a level of comfort but were at the same time impediments to change. Negative orientation toward being overweight and to the experience of attempting to lose weight led to frustration, fear, depression, guilt, despair, and a sense of failure. Negative feelings towards the process of weight loss itself aligned to both dieting and exercising. Dieting was perceived as anti-social and isolating. Exercise was perceived as difficult to achieve for reasons including the time it required, lack of motivation, finding an appropriate activity or venue, cost, lack of confidence, having no energy, and being pushed too hard. The impact of internal impediments could be overwhelming, with no clear way out, perpetuating the sense of battle (Theme 1).	*I think if I have got the willpower to do a whole lot of other stuff that I do which absolutely takes determination day in and day out. Why doesn’t it work for the food thing? And I sort of wonder what the psychology of willpower is. (Female 66a)* *The emotional thing is my biggest battle it’s that whole I get upset so I turn to food, you know I get angry I turn to food, I am happy I turn to food. . . . I need someone to help me control those emotional links that I have with food, I need someone to teach me other strategies when I am feeling down. . . . I might lose weight but then I come off the diet so to speak and go back to normal eating. (Female 32)* *…it is in my mind all the time, that’s in the freezer… and I will go out and I will have one, a little cone and then sit in front of the TV and think, oh that was lovely and back out and I have to have another one…and then feel quite ill and guilty. . . . Whenever I’m in hospital I eat like a horse ‘cause everyone thinks the hospital food is so awful but I just eat everything they put in front of me yeah um cause I, I don’t know, I feel comfortable. (Focus group 2, Female 61)* *I find myself saying negative things about myself all the time. … I don’t like fat any more than anybody else, in fact I probably like it less than anybody else.. . . I hold on to the weight, as if I have to make myself bigger to protect myself or something, which is exactly the opposite of what I want to do. . . . Losing weight to me has been a very important goal and I just cannot achieve it. (Female 56)* *We’ve been educated about food and what we should and shouldn’t be eating and doing, but we still do it, so there is an element of guilt. (FG3, Male 69)* *. . . most of my eating most of my life has been comfort eating and it has been emotional. (FG3, Male 42)* *I don’t feel like I’ve got any control of anything right now I’m not in control of myself. . . . I can’t get motivated, I can’t do anything right now I am just so physically and mentally exhausted. . . . Eventually I will just drop dead of a heart attack and I’m thinking well you know maybe that’s an easier way out for me, that’s an awful way to be thinking. (Female 61b)*
Relational impediments	Relations with others were often not helpful and sometimes an impediment. These influences could act as a barrier to positive progress. Health professionals we important in providing information and support, however experiences were varied. Negative experiences were not uncommon, resulting from an apparent lack of understanding of the complex experiences relating to obesity. Insufficient attention to the mental health or the psychological aspects of obesity by health professionals limited the usefulness of these interactions. Conflicting advice, not enough information, or a lack of follow through were further impediments with inadequacies related to a lack of time, an inability to adequately discuss needs or give direct advice, a focus on bariatric surgery as a silver bullet solution, and lack of familiarity regarding other resources for weight loss management. Knowledge deficits were apparent: a lack of knowledge about health professional support; a lack of knowledge or understanding about how to achieve weight loss; and contradictions with conflicting messaging from multiple sources.	*…he said it’s a mathematical equation, you put energy in, you expend energy and the result is a loss in weight, that’s it, simple….. (Female 56)* *It’s amazing how you can be completely invisible and enormous at the same time. (Female 66a)* *Oh I have had a skinny doctor too who just kept bagging me every time I went in there for being fatter than last time. (FG2, Female 45)* *. . . you get some information from here and some information from there but you never know if it is accurate and you can try going down one path and find it a complete failure and then you can get another bit of information and try that for a while. (FG2, Female 45)* *… still hadn’t heard anything so in the end I contacted her a fortnight later and I got told that she had been overseas for a week, so at this point I am totally disillusioned because I didn’t know whether or not my food diaries were right or wrong or what I was eating was right or what I was eating was wrong… (Female 42)* *. . there is no-one to walk with, my girls work full-time and they’re all tired so I’m in a pickle . . . I just find that when you live on your own, you seem to put everything in the too hard basket. (FG1, Female 69a)* *…you hear this about cutting your carbs down and you will be alright and then someone else says, no, no you cut your fat down and then someone else says you cut your sugar down, I don’t know maybe you are supposed to cut them all down…(FG2, Female 61a)* *. . . for the last 30 years every doctor I ever tried to speak to about being depressed, straight away here’s some Zoloft... I don’t want to take that I want to deal with what’s going on. . . . ...he can be quite dismissive of things you know. (Female 61b)* *It is hard to get access to personal trainers, it is hard to get access to dietitians at the hospital, there is an enormous wait to get into those things and you tend not to be able to get, my GP wouldn’t know where to start. (FG2, Female 45)*
Systemic barriers	Negative societal perceptions and biases towards people who are overweight were felt widely. Participants were aware of others’ attitudes and judgements about not being good enough, not trying hard enough, being excessive eaters, lovers of unhealthy food, lazy, stupid, ugly, dirty, and not deserving of respect. For some isolation was problematic, not having someone to be there, to provide motivation, allowing negative behaviours to perpetuate. Barriers to accessing services, community clubs, and events which could serve to promote health occurred, sometimes these barriers were practical (e.g. financial, lack of transport, responsibility for children) and sometimes they related to a negative culture within society and within organisations towards obesity. Access to health services remained an enigma, leaving participants wondering how to find the help they needed	*If you are fat, you are stupid and it drives me bananas and there is that automatic you know, silly dumb woman. (Female 66a)* *You have McDonalds and some you know yobbo will say ‘what are you having Maccas you fat so and so’. (FG3, Male 42)* *Oh yeah you do feel like you are being looked at when you are walking around with a shopping trolley, you do. . . . I want to exercise, I really do, but I want to feel comfortable doing it somewhere and without a lot of funds I don’t know of anywhere I can do it for free in an enclosed, safe place. (FG2, Female 45)* *See a mum with two kids, with depression, how can she possibly join the gym that only has sexy, beautiful girls on the poster. (FG1, Female 42)* *…families are using McDonalds as it’s twenty bucks and you have fed your whole family, whereas if you go and buy fresh fruit and vegetables, which I actually do, it costs a hell of a lot. (FG1, Female 55)* *…when you haven’t got transport it is very hard to get somewhere to join in. (FG1, Female 69)* *With the GP for example, they don’t have time, they have a waiting room full of people. (Female 56)* *When going to someone else’s place when you only eat a small portion and they get really offended and I get really worried about it. (Female 66a)*
Theme 3 Positive re-orientation *“I walk every day and eat breakfast obviously something is going right at the moment; I am losing a bit of weight.” (Female 48)* Subthemes associated with positive reinforcement followed a potentially chronological process which begins with a shift to an empowered orientation, leading to positive activity, and resulting in behavioural reinforcement. As we have seen from previous themes, this linear description is too simplistic, and the complexity previously described prevails, however an understanding of this process is useful in formulating knowledge to facilitate the process.		
Empowerment and motivation	A positive change in mindset was the first step towards acting. Motivation played an important part in this change, intrinsic and extrinsic, often the two occurring simultaneously. Improved physical and psychological health, wanting to habitat a different shaped body, wanting a fuller engagement in family activities and to be there for children, were important motivations. Social connection could act as motivation - the focus groups themselves motivating, but ultimately there was a recognition that intrinsic motivation was key. Knowledge and understanding the benefits of healthy eating and knowing that there are long-term effects of eating the wrong foods was also a condition to promote empowerment. Knowledge as insight was expressed as feelings, intuitive knowledge or known fact. Ownership of health was understood as personal responsibility but required knowledge of what it is preferable in terms of actions. Overwhelmingly participants felt more empowered when their whole circumstances and health management were considered and pro-actively addressed by health professionals. This helpful interaction included the health professional being honest, respectful, listening and addressing the difficult issues as well as referral to other health professionals as required. Individualised management and planning for achieving weight loss strategies was considered necessary with the need for psychological support and adequate information. Realisation that there had been a change in thinking and attitude toward making change occurred sometimes as a pleasant surprise.	*Well my doctor said if I don’t get the weight down I’m going to become a diabetic. (FG3, Male 63)* *. . . you see somebody that you know you know that’s had a battle and you see something good come out of that and it kind of inspires you a little bit. (FG3, Male 34)* *I realise that I was eating too much of the wrong kind of things. . . . But the buck stops with me . . . you have to get up and just decide well today is the day I am going to change my life. (FG1, Female 55)* *I know a lot of the solution has to come from me. (Female 32)* *…it’s not just for me it’s for my kids I want to be able to go and do things with them, I want to be here for a long time. (Female 59)* *I have been exercising, I feel as though I don’t want to eat all this horrible stuff. I am starting to get this mindset about, hey come on you are doing this now you have to keep it up with the food. (FG2, Female 61)* *I know too with our children we have [to] do right so that they learn. (FG1, Female 42)* *For me the best idea would be for each person to discuss, with the right, appropriate person full of information, is what do you want, do you want general fitness, do you want flexibility, do you want weight loss… and tailor something to that person. (FG2, Female 45)* *I thought somebody was at last starting to take a holistic look at the problem, rather than at specifics [agreement] and I think um the approach between the dietitian and the psychology, psychological approach is the right approach. (FG3, Male 71)* *Mainly for me at the moment it’s the why I do it and how to then cross that bridge in to stopping that behaviour. . . . I need someone to help me control those emotional links that I have with food, I need someone to teach me other strategies when I am feeling down. (Female 32)* *. . . with us four just being here it seems to motivate me to talk about things, but then I go home and I am back to square one again, no one to talk to. (FG1, Female 69a)*
Positive action	Small but realistic lifestyle changes were important in achieving change and enabled positive action. Learning that it was counterproductive to exclude foods which provide pleasure was important. Becoming more physically active relied on finding the right activities in a supportive, non-judgemental environment. Support in understanding and acting on the psychological aspect of weight and the contributing behaviours enabled a holistic approach. Finding a supportive environment with the right people to provide support helped to facilitate positive action. Finding others who had shared experiences, with a realisation that it was not necessary to go through change alone, provided a context to facilitate change.	*I answer the phone standing up now… I get up, I stand up and whether it is a mobile call or the house phone I stand up and that might only be for 5 minutes or 10 minutes but it is 5 minutes or 10 minutes that I am standing up which is better than sitting down. (FG2, Female 45)* *. . . never deny yourself of anything… yeah it was a matter of moderation. . . . if I need to eat a chocolate bar to be happy well I will eat a chocolate bar to be happy. (FG1, Female 42)* *I used to have two big teaspoons of sugar in my coffee every day but I have cut down to a small teaspoon. The first two mornings were ‘ewh’ - it was like poison, but after that I persevered and now I am used to the one … ‘I’ve cut down on my sugars, I’m a good Mum’, I was quite pleased with myself. (FG1, Female 69)* *. . . .it’s probably the first time for me in my life that I’ve ever really concentrated on doing something for me.” (FG3, Male 39)* *Yes, they have all these trainers there to put you on the right track … I went yesterday and I went last week but no-one, it seems to me that no-one is judging you. Like, some are big, some are little, but we’re all there for the same reason. (FG2, Female 61)*
Behavioural reinforcement	Positive activity was particularly successful when associated with a learning that tackling the problem head on through behaviour change can lead to positive emotions and result in a sense of satisfaction at doing something for one’s self. Behavioural reinforcement further promoted positive re-orientation through feeling good, physically and psychologically, triggered for example by a realisation that some weight loss had been achieved, noticing that clothes fitted better, enjoying shopping for clothes, and feeling physically fitter or healthier. Positive reinforcement from others also occurred. Receiving compliments and significant people offering to support the desired behaviour changes.	*. . . nobody mentions how wonderful you will feel if you just do that, you know, it’s a funny sort of thing. They will say “you will be better for it” but it’s not quite the same as you will really enjoy doing … something really small like how you feel after a meal if you have eaten such and such, and it is the pleasure of particular things, see it is all the things that are good for you. Nobody actually says, you will actually really enjoy the physical thing of eating. (Female 66a)* *I’ve been walking a lot… I’ve been walking around the block for 30 minutes … and then I go and walk the big block and I love it. (FG2, Female 55)* *I felt much better you know I could buy nice clothes which I couldn’t before, um and I can’t now, and um and I enjoyed going shopping and I used to hate going shopping and I hate going shopping now, um and you know I just felt more positive about myself in general. (Female 61b)* *I think I am improving in stamina, I’m like when I first started ballroom dancing I could only do a little bit and then I was, puff, puff, puff and now I can go to the social dance and dance most of the night . . . That sort of boosts your self-esteem up straight away because years ago I used to do that sort of thing, dancing every week. (Female 56)*

### Integration with Lifeworld led care dimensions

It was an explicit aim of this study to identify dimensions of the lived experience of obesity, those themes and/or dimensions which might either impede or facilitate access to and engagement in weight loss intervention. To achieve this we explored relationships between the themes and subthemes disclosed through thematic analysis, and the lived dimensions of the lifeworld according to LLC: temporality, spatiality, intersubjectivity, embodiment, identity and mood [30].

**Fig. 2:**
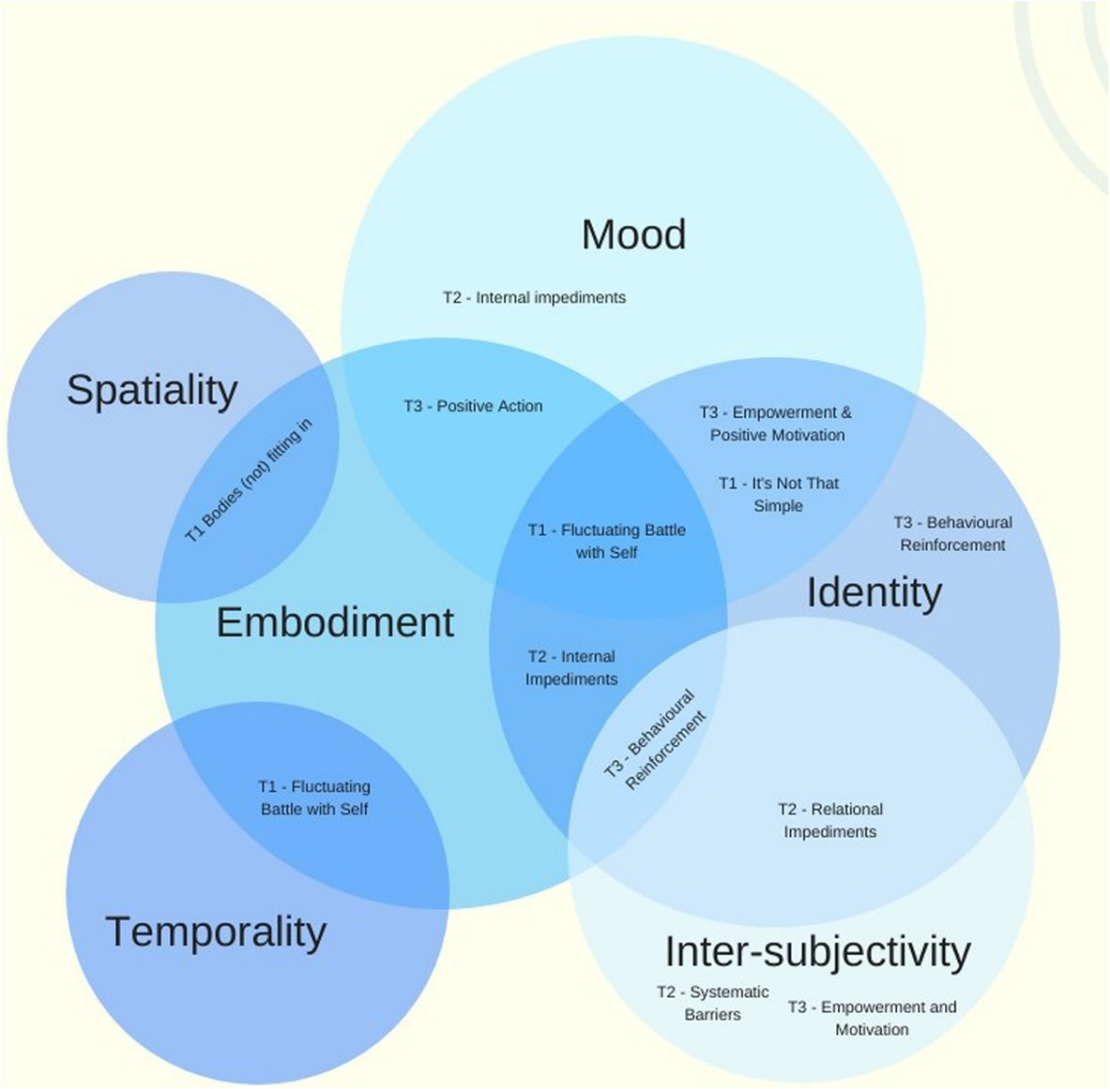
Integration of Subthemes and Lifeworld Dimensions. Figure Legend: T1 – Complexity and Battle. T2 – Impediments. T3 – Positive Reorientation

The three Primary Themes (Complexity & Battle, Impediments and Positive Re-orientation) and their subthemes have been mapped and grouped in relation to lifeworld dimensions as categories of lived experience (Fig. 2, and Additional File (item 2) for a detailed explanation of the relationship of subthemes to LLC dimensions). All subthemes were able to be related to one or more of the LLC dimensions, indicating the complexity and inter-relatedness of the themes, and their relevance to LLC.

### Polarised dichotomies

**Fig. 3:**
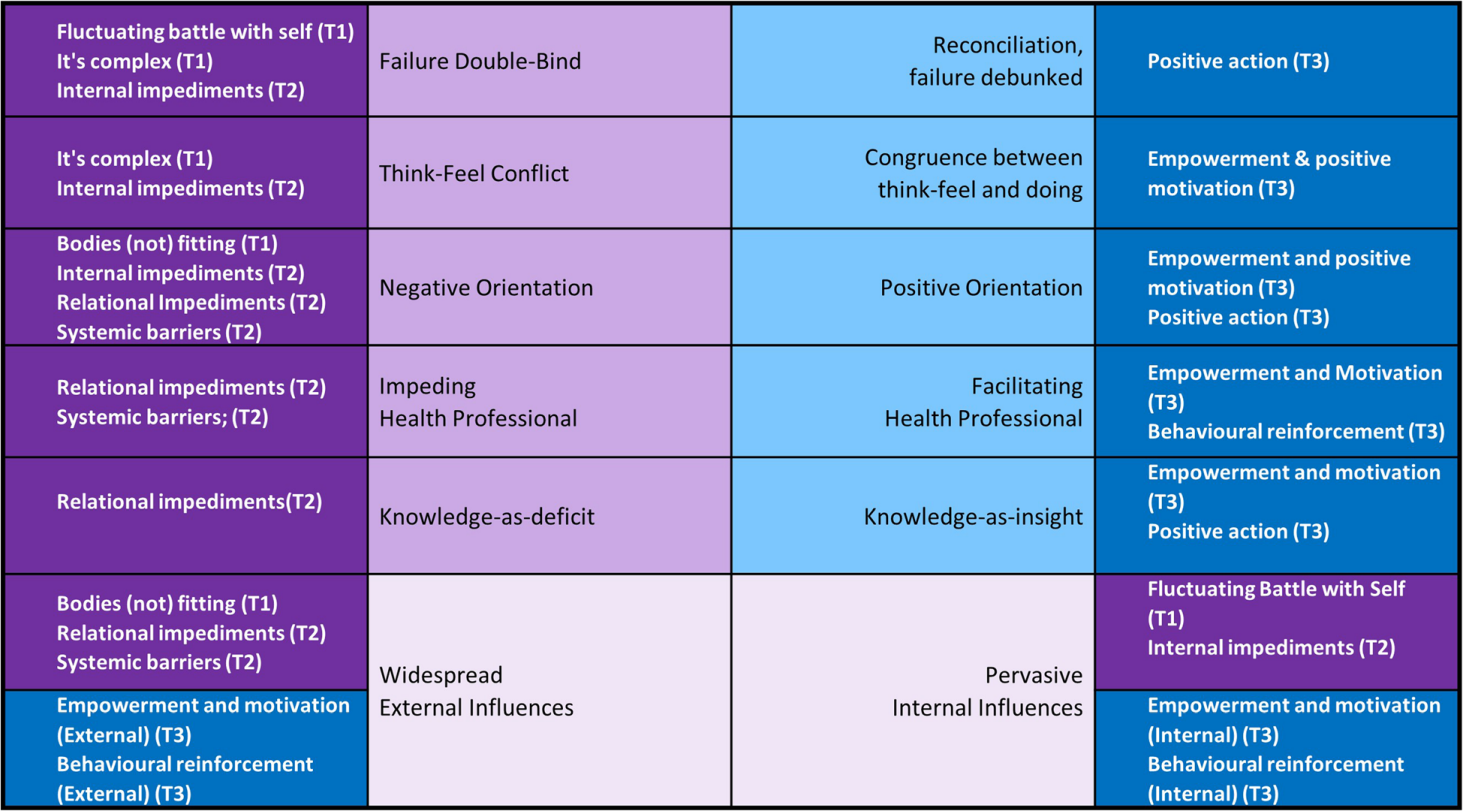
Representation of the integration of dichotomies with themes and subthemes

**Fig. 4:**
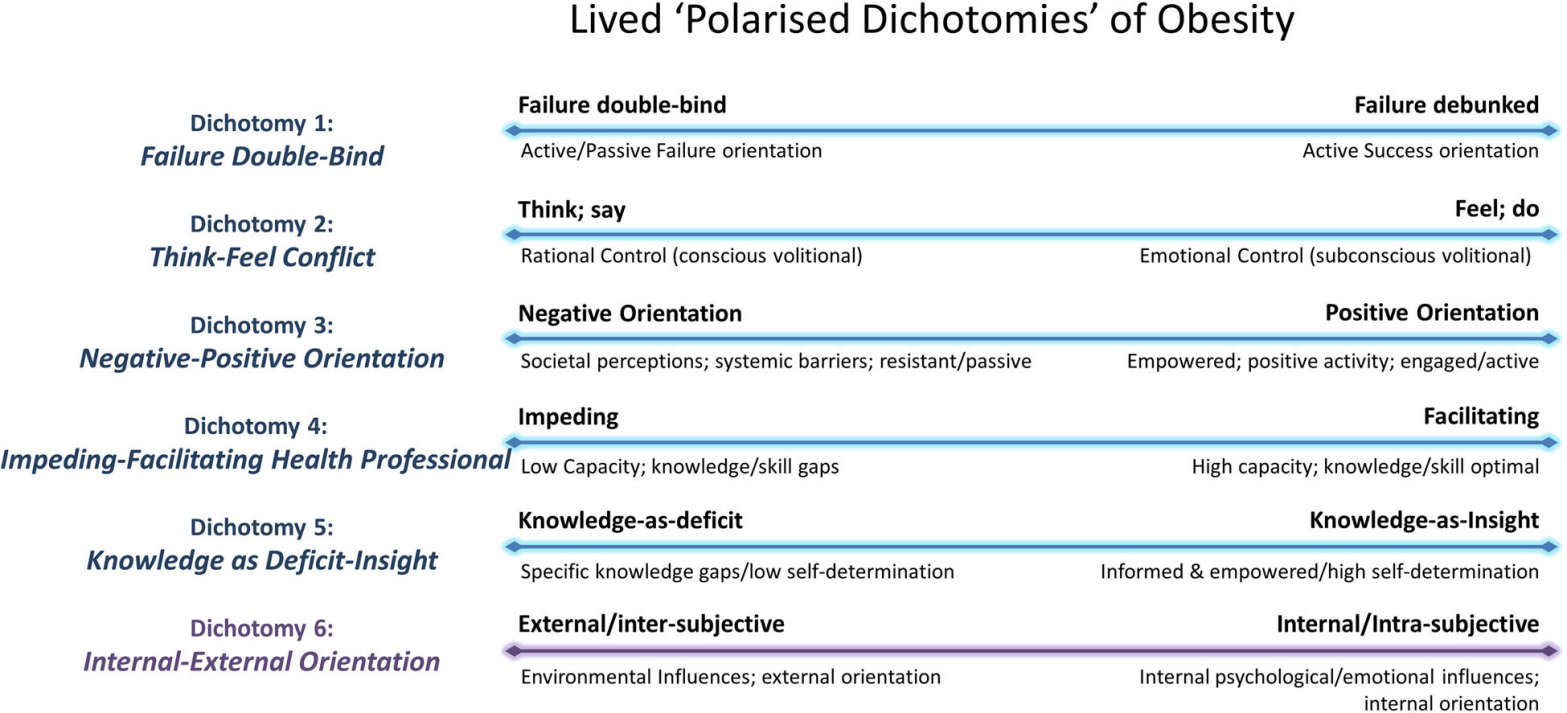
Lived Polarised Dichotomies of Obesity (LPDO)

Further scrutiny of the themes and subthemes in the context of the LLC dimensions led to the determination of a number of dichotomies representing the lived experience of obesity. These dichotomies represent a complex integration of the themes and subthemes (Fig. 3) and emerged as it became apparent that themes represented polarised positive and negative experiences of the same notion. Six dichotomies occur on a continuum between two extremes, with an individual falling somewhere along each continuum at any given time (Fig. 4).

#### Dichotomy 1: failure double-bind

Participants identified a complex psychological double-bind which resulted in confusion and/or a static behavioural arrest. They described themselves as caught and vacillating between actively attempting weight loss and failing, and in contrast not actively attempting weight loss due to anticipated failure. This pattern of evidence-building on both ends of a failure continuum undermined attempted weight loss and reinforced expectations of failure leading to a sense of inevitability, hopelessness and self-fulfilling prophecy around weight loss efforts and diminishing self-efficacy.

#### Dichotomy 2: think-feel conflict

A conflict was consistently identified between what participants cognitively think and express in language, and what they emotionally feel and express in behaviours. A conflict between rational and emotional forms of logic, where rational logic dictates what one articulates, is in tension with an equally valid fluid, embodied and emotional logic which dictates what one does. This indicates a conflict between what one thinks one should want or do, and what one wants at a more emotional and often less conscious level. For this population, where emotional motives often govern behaviour, the conflict results in a behavioural homeostasis and subsequent self-perpetuating weight gains.

#### Dichotomy 3: negative-positive orientation

The need for a reliable mechanism for re-configuring negative orientation into positive re-orientation was identified. It spanned polarised themes such as negative vs positive behavioural reinforcement and influence of others which could constitute either a negative or positive factor. Empowerment and positive activity were polarised with societal perceptions and systemic barriers, with explicitly linked implications for weight loss success and failure respectively.

#### Dichotomy 4: impeding-facilitating health professional

Health professional interaction was a subtheme of positive re-orientation with a polarised tension in the impediments theme. This polarised tension exists in the Inter-subjective lifeworld dimension reiterating that the capacity of health professionals is experienced as either a positive reinforcing factor facilitating access/engagement, or as a negative factor impeding access/engagement.

#### Dichotomy 5: knowledge as deficit-insight

Knowledge deficits as an impediment was contrasted with knowledge as insight as positive re-orientation, meaning knowledge could be experienced as either a positive empowering force, or as a negative undermining factor. Importantly, knowledge was identified as a variety of forms, such as the straightforward content-based knowledge required to make informed food choices, and in subtler and potentially undermining forms of evidence gathering. Also indicated is that absence of necessary knowledge is often a causal factor in behavioural stasis, while the insight gained through knowledge informs and empowers behavioural shift.

#### Dichotomy 6: permeating dichotomy: internal-external orientation

The polarised dichotomy which permeated, complicated and mediated the other five dichotomies was that of internal, intra-subjective factors influencing weight, contrasted with external, inter-subjective factors influencing weight. This dichotomy highlights a polarisation between the lifeworld dimensions of Identity (as intra-subjectivity) and inter-subjectivity. The permeating dichotomy of Internal/External, with features such as psychological flexibility and emotional motivations for weight retention, saw a high degree of contrasting psychological factors at play. This is a complex domain where orientation is heavily influenced by experience of other, in either positive or negative ways, and intimately relates to more subtle polarisations, such as that between compulsivity and empowerment in the identity (intra-subjective) dimension.

## Discussion

Complex relationships have emerged between the lived experience of our participants and the fundamental dimensions of the Lifeworld-led Care (LLC) [30] which have implications for weight loss intervention. The lifeworld dimensions of inter-subjectivity, mood, embodiment and spatiality are complex and important, but the dimension of identity exhibits the most rich and complex interplay of themes and subthemes across multiple lived dimensions.

‘Fluctuating battle with self’ encapsulated the four lifeworld dimensions – embodiment, identity, temporality and mood. This subtheme re-presents complexity as an interplay of the physiologically lived (embodiment), the psycho-socially lived (identity) and the emotionally lived (mood) as a perpetual internal struggle (battle with self) - the “messy ball of wool”. This struggle and complexity expressed in our findings is consistent with others [26, 41], whereby gaps and tensions between knowing and doing in relation to food habits, and the fluctuations within these experiences, create barriers to change. Objectification [42] quietly serves a de-humanising function for the person who lives their ‘weighed and measured’ condition. Such compromise to personhood is clearly apparent in the intersubjective dimension incorporating subthemes found in the impediments theme, such as perceptions, systemic barriers, and influence of others.

Lifeworld dimensions of spatiality and temporality are evident in the complexity and battle theme. How bodies ‘fit’ in daily living is exemplified by participants describing prolonged, compromised embodiment over time. Confidently sitting in a chair, buying clothes that fit, painting toenails, are all taken-for-granted activities for people without weight concerns, but complex battles for people who do.

LLC’s macro-agenda for the humanisation of care [27], applies directly to the individual human predicament, acknowledging the limitations of the lived experience while being open to possibilities within it [28]. This entails explicitly dwelling in paradox and inhabiting existential tensions with life as it is, while also finding whatever avenues persist for living forward; referred to by Galvin and Todres [30] as dwelling mobility. In the situation of a person whose existential possibilities are compromised by both their embodied, socially constructed and internalised weight, it can be profoundly complicated to identify inherent possibilities for living forward in new ways. One interpretation of the desire for bariatric surgery, for example, is in its potential to facilitate a new avenue for living forward.

Appreciating the lived experience of obesity in more subtle and complex ways allows barriers as well as possibilities to be identified “… for a more humane arrangement of things” ([42, p. 81]), consistent with LLCs emphasis on the re-humanisation of care. This need for greater patient-centred care is evidenced by the representation of Lifeworld dimensions inherent in the data. The polarisation of the health professional interaction as either a positive helpful facilitating influence or an impediment to engagement is one specific area for improvement. Translating this into the lived experience of care, a more humane approach can be as simple as resisting systemic pressure to quantify obesity as a condition, and thereby reducing the human being to kilograms or BMI [17].

Gadamer’s notion of health as well-being, whereby a person is ready for new things and feeling carefree [42], stands in stark contrast to the lived experience of our participants, both in terms of their shifting ability to move forward, and their lived experience. Negotiating goals of care is hindered when health professionals and care systems are limited in their understanding and application of qualitative dimensions [43]. The multidimensional approaches of Thomas et al. [5] from a decade ago is important in creating a broad framework but lacks the detail on how to address specific needs of individuals.

### Clinical implications for practice and education

‘Dichotomous thinking’ has been reported previously [26, 41] as one subset of multiple barriers in the obesity experience. Rogerson et al. [26] describe weight loss as a journey punctuated by experiences that assist or impede progress, and encourage further research into strategies that mitigate, among other things, the challenges of dichotomous thinking. Christiansen et al. [41] describe the struggle between knowing and doing, aligning with our dichotomy of knowledge – deficits and insights. We have presented the entire dataset of themes and subthemes across Lifeworld dimensions as ‘Lived Polarised Dichotomies of Obesity’ (LPDO), a significant finding of the study and a way of uniquely carrying forward the complex and interacting meanings identified in the data. The dichotomies occur on a continuum between a negative and a positive extreme. The negative end of these polarisations is associated with behavioural arrest, diminished self-efficacy and psychological adjustment, behavioural stasis and weight retention/gain. The positive ends of these polarisations exhibit more informed, empowered and adaptive behaviour, coupled with greater efficacy, psychological flexibility and resilience, suggesting clear areas of prioritisation for the development of weight management services and interventions. The polarised dichotomies can provide valuable insight into facilitating and impeding factors in weight management intervention, and provide a resource for clinicians, service designers and policy makers to champion the re-humanisation of health care for this complex and endemic population and apply them to practice. A clinical approach can be taken to meet the self-identified needs of the population (Table 3).

**Table 3:** Recommendations for clinical application of dichotomies

**Failure Double-Bind**	
**General principle**	
• Most people with weight problems have a history of attempting weight loss strategies resulting in complex feelings of failure and learned helplessness.	
**Psychoeducation**	
• ‘Re-engineer’ the construct of ‘Failure’ as a ***double-bind*** (try & fail = failure (active); don’t try = failure (passive)). Moving from active failure to passive failure, yoyo weight loss/regain and behavioural arrest. • Re-configure the construct of ‘success’ as ***non-quantified*** outcomes with emphasis on qualitative markers (improved fitness/mobility, a changed relationship with food).	
**Behavioural Health Consultation**	
• Debunk the notion of failure through modelling of weight-language • ***De-emphasise*** numerical weight-loss goals and reframe goals in qualitative terms. • Positive reinforcement of the value of all attempts to change health behaviours and acknowledgment that unsuccessful attempts do not constitute failure • Reduce learned hopelessness, improve self-efficacy, and address behavioural arrest.	
**Think-Feel Conflict**	
**General Principle**	
• Rational control vs emotional control is central in volitional eating. Rational control manifests in what people think and say, while emotional control manifests in food choice and eating behaviour. Framing this conflict in terms of ‘control’ facilitates empowered responsibility for food choice and eating behaviours.	
**Psychoeducation**	
• Debunk the control conflict between ***conscious volitional*** eating and ***subconscious volitional*** eating – eating behaviour is ***always*** volitional behaviour • Identify and examine the internal conflict created by the gap between thinking/wanting and feeling/behaving	
**Behavioural Health Consultation**	
• Complete an inventory of weight-related thoughts, feelings, and behaviours, identifying key conflicts • Develop alternative weight-related health behaviours to reduce conflicts • *Identify ***weight-retention motivations (*e.g.*self or relationship-sabotage)*** and refer for specialist intervention if required	
**Negative-Positive orientation**	
**General principle**	
• Explanatory Style is a key element in the ways people interpret environmental factors, social experience and their own behaviour. A negative orientation closes the individual to possibilities for change and perpetuates learned helplessness, while a positive orientation opens the individual to new possibilities despite the lived constraints and limitations of circumstances	
**Psychoeducation**	
• Facilitate awareness of the role of psychological predisposition to negative or positive orientation • Raise awareness of the role of predisposed orientation in perpetuating think/feel conflicts	
**Behavioural Health Consultation**	
• Facilitate improved capacity to self-identify negative orientation, its underlying causes and implications	
• Expose and debunk negative orientation, facilitating positive orientation.	
**Impeding-Helpful Health Professional**	
**General principle**	
• Health professionals either facilitate or impede access and engagement in weight-related behaviour change, depending on their level of knowledge and skill with weight-related intervention. Capacity building for health professionals to understand and engage people in weight-related health care is vital.	
**Psychoeducation**	
• Education and training in polarised dichotomies of obesity for front line health professionals to reduce knowledge gaps and increases the capacity to reinforce knowledge-as-insight.	
**Behavioural Health Consultation**	
• Training, education and resourcing of frontline Health Professionals in a responsive, integrated, ‘psychodietetic’ approach to weight-related intervention to build optimal response to weigh-related presentations [43]. • Emphasis on collaborative and integrated multidisciplinary support.	
**Knowledge as Gaps-Insight**	
**General principle**	
• Knowledge is required to inform food choice and eating behaviour change, it cannot be assumed and should be provided in an iterative and communicatively competent manner – increased knowledge/autonomy/ relatedness leads to increased self-determination and intrinsic motivation.	
**Psychoeducation**	
• Providing adequate, consistent and iterative advice to facilitate insightful, reflective knowledge into motives for self-sabotage, weight gain and/or weight-retention. • Identify and address psychodietetic knowledge gaps to empower sustainable food choice and eating behaviour change (e.g. emotional triggers, label-reading, viable ‘swaps’).	
**Behavioural Health Consultation**	
• Appreciation that eating behaviour is motivated and reinforced behaviour. • Ensure knowledge is broad and holistic and entails self-reflective understanding of emotions, weight-retention motivations, and potential sabotage. • Development of alternative strategies for self-soothing as behavioural substitutes.	
**Internal-External Orientation**	
**General principle**	
• The permeating, fundamental dichotomy of ‘internal/external’ specifically identifies the role of intra-subjective preoccupation (e.g. habituated, ruminative, internally oriented self-appraisal or self-reflection) as a potentially causal factor in many eating behaviour profiles.	
**Psychoeducation**	
• Facilitate improved capacity to regulate orientation either ***intra***-subjectively, ***inter***-subjectively, or ***externally***, through behavioural techniques and attention training strategies.	
**Behavioural Health Consultation**	
• Facilitate insight into, and shift of, habituated ***intra***-subjective orientation to consciously directed ***external*** orientation. • Facilitate insight into and decrease in weight/eating-related ***intra***-subjective monologue Facilitate increased capacity to self-regulate emotion with non-food strategies (e.g. relaxation or substitution strategies). • Facilitate increased capacity to regulate attention on benign stimuli (e.g. breath, sensation). • Cognitive modification of counterproductive weight-related attributions.	

Findings of emotional alteration and a need to develop a deeper understanding of perceptions and experiences of people with obesity are consistent with recent findings from a Norwegian study [44]. There are calls for a holistic, comprehensive, multidisciplinary approach to obesity management and research [7, 17, 45], and an identified gap in the availability of tools which provide practitioners with a more structured approach to management and which allow a more authentic interaction [43]. Findings from this study suggest that any innovative intervention or service system design should strategically address the six identified dichotomies of the lived obese experience. Translation of this approach to practice requires future investigation of how the model can strengthen existing interventions and provide practitioners with additional tools.

## Conclusion

If living with obesity is a ‘messy ball of wool’ as described by our participants, the dimensions of the lifeworld are different coloured strands, which at the very least offer us a more textured grasp of the whole, and perhaps some places to begin the unravelling. The identification of which lifeworld dimensions were most significant in lived experiences of obesity provides important insights for providers of weight loss intervention. We suggest that obesity manifests as constraints and challenges across six polarised dichotomies, active in the lived experience of obesity. Each dichotomy has direct implications for either facilitating or impeding access to and engagement in effective intervention. If these dichotomies are better understood by practitioners as well as students, we may progress the untangling of the complex experience of obesity. We advocate a radical reconceptualization of obesity from a quantification of the individual, to a more respectful, humane, compassionate and utilitarian conceptualisation of the phenomenon, encompassing the polarised dichotomies revealed in this study. Working with weight-related problems across these six explicitly identified lived dichotomies offers a clear guide to developing innovative, effective clinical practice interventions that individuals want, need and will feel able to access.

### Limitation

The study population was white Anglo-Saxon from a developed country, external validity outside this population group should not be assumed.

## Supplementary information

**Additional file 1.** The additional file includes the interview schedule (Item 1), a list of subthemes and raw evidence of them from transcripts (Item 2), and a detailed explanation of the relationship of subthemes to Lifeworld Led Care dimensions (Item 3).

## Data Availability

The datasets generated and/or analysed during the current study are not publicly available. Due to their qualitative nature, the data are potentially identifiable. Extended examples of quotes from the data which represent the themes and subthemes identified in the analysis have been made available in the additional file. The full dataset may be made available by the corresponding author upon reasonable request.

## Abbreviations

FG1: Focus group 1
FG2: Focus group 2
FG3: Focus group 3
LLC: Lifeworld Led Care
LPDO: Lived Polarised Dichotomies of Obesity
