# Supplementary material for: A “messy ball of wool”: a qualitative study of the dimensions of the lived experience of obesity
Source: BMC Psychol. 2020 Jun 25;8:67. doi: 10.1186/s40359-020-00416-2 (PMC7318440; doi:10.1186/s40359-020-00416-2)
Supplement: Supplementary file 1 — Additional file 1. The additional file includes the interview schedule (Item 1), a list of subthemes and raw evidence of them from transcripts (Item 2), and a detailed explanation of the relationship of subthemes to Lifeworld Led Care dimensions (Item 3). [file 40359_2020_416_MOESM1_ESM.docx]

**Additional file**

**Item 1**

Table. Interview schedule

| - **What does it mean to you to be overweight? How does it affect you emotionally and physically?** - **What is your understanding about your eating style? Where did that style come from?** - **How important is it to you at this time to make changes that will lead to improvements in your health?** - **What are the things that influence you to make changes?** - **Are there any pre-requisites for change?** - Have you made healthy changes in the past? - What is it that has helped you do this in the past? - What programs, services or health care professionals have helped you in the past? How have they helped? - What other people in your life have helped in the past? How have they helped? - **Why have you slipped back into poorer habits?** - **How confident do you feel in making healthy changes? What would make you feel more confident? What would make you feel less confident?** - **What services do you envisage would help in the future?** |
| --- |

**Item 2**

Table. Representation of Lifeworld Dimensions in the raw text through mapping with themes generated from thematic analysis.

| **Lifeworld dimension (Galvin & Todres, 2011)** | **Representative themes and subthemes (see Figure 2 in main text for visual representation)** |
| --- | --- |
| **Temporality:** The human experience of time, not just as a quantitative measure but also in a qualitative way, for example in possibilities and changes in condition over time | In the lifeworld dimension of Temporality, just one subtheme was identified: ‘Fluctuating Battle with Self’. This theme was also identified in conjunction with the lifeworld dimension of Embodiment, indicating obesity as a protracted experience of embodiment over time; a prolonged and episodic sense of embodied struggle with weight.  The relatively low representation of themes associated with Temporality could indicate a static experience of time in the lived experience of obesity, and that Temporality is not identified as a lifeworld dimension which either facilitates or impeded access to/engagement in weight loss intervention. |
| **Inter-subjectivity:** How we are in the world with others; understanding self through how our lives take place within a social world | In the lifeworld dimension of Inter-subjectivity, the themes of Impediments and Positive Re-orientation were represented through seven different subthemes. The subthemes of ‘Empowerment and Motivation’, ‘Behavioural Reinforcement’, and ‘Systemic Barriers’ highlighted the importance of the health professional and the health system as either facilitator or impediment to access/engagement in intervention. The subtheme ‘Relational Impediments’ is also integrated with the Identity dimension, and ‘Behavioural Reinforcement’ spans the Embodiment dimension. This indicates a significant and complex interplay of these subthemes across multiple lifeworld dimensions and provides important insight into the lived complexity of the Inter-subjective dimension. |
| **Embodiment:** The concrete here, the lived body; how we ‘bodily’ live in relation to others | All three Primary Themes are represented and significantly, there was no subtheme that was related to embodiment alone. Embodiment interacts with all five other dimensions. The subtheme ‘Fluctuating Battle with Self’ was also related to the Temporality, Identity, and Mood dimensions; and ‘Internal Impediments’ were associate Embodiment with the Identity dimension. ‘Positive Action’ was also associated with the Mood dimension, and ‘Behavioural Reinforcement’ with Inter-subjectivity and Identity dimension, indicating the positive reinforcing influence of ‘other’ in relation to the lived embodiment dimension. |
| **Mood:** Lived experience is shaped by mood, which penetrates and shapes the spatial, temporal, intersubjective and embodied horizons. A messenger of the meaning of situations. | All three Primary Themes are represented. There are complex interactions of these themes and subthemes with the lifeworld dimensions of Identity and Embodiment. The subthemes of ‘It’s Not That Simple’ and ‘Empowerment and Positive Motivation’ is shared with the Identity dimension, and ‘Fluctuating Battle with Self’ interacts across all three of Embodiment, Identity and Mood dimensions. ‘Behavioural Reinforcement’ identifies a relationship across the Mood and Identity dimensions of the lifeworld, while ‘Internal impediments’ (from the Impediments theme) relates to the Mood dimension alone, suggesting that negative orientation is experienced as an intra-subjective mood state which impedes access and/or engagement. ‘Positive Action’ relates to both the Mood and Embodiment dimensions. |
| **Spatiality:** Existence in terms of distance or closeness to the places and things which have meaning, not just physically but also emotionally | Lifeworld dimensions of Spatiality is evident in the Complexity and Battle subtheme ‘Bodies (not) Fitting’, where participants describe prolonged, compromised embodiment over time. Taken-for-granted activities for people without weight concerns become complex battles for people who do |
| **Identity** | With a total of seven subthemes spread across all three Primary Themes, the Identity dimension of the lifeworld emerged as the most complex dimension of lived experience for our participants. As a singular dimension, Identity was related with ‘Behavioural Reinforcement’ indicating the value of positive re-orientation on identity in the obesity battle. ‘Empowerment and Motivation’, ‘Fluctuating Battle with Self, and ‘It’s not that Simple’ revealed relationships between the Identity and Mood dimensions, and as a cluster these three subthemes suggest a polarisation where each subtheme can constitute either a facilitating or impeding experience.  The subthemes of ‘Fluctuating Battle with Self’, ‘Internal Impediments’ and ‘Behavioural Reinforcement’ provide evidence of a significant relationship between the lived dimensions of Identity and Embodiment. This suggests embodied identity is lived as a complex internal battle between Habituation and Compulsivity. ‘Relational Impediments’ contrasting with ‘Behavioural Reinforcement’ demonstrate a relationship between the lived dimension of Identity and Inter-subjectivity. |

Galvin, K., & Todres, L. (2011). Kinds of well-being: A conceptual framework that provides direction for caring. *International journal of qualitative studies on health and well-being, 6*(4), 10362. doi:<https://doi.org/10.3402/qhw.v6i4.10362>
